# A Dual-Axis Rotation Scheme for Redundant Rotational Inertial Navigation System

**DOI:** 10.3390/mi14020351

**Published:** 2023-01-30

**Authors:** Ting Zhu, Lifen Wang, Tao Zou, Gao Peng

**Affiliations:** 1 School of Automation, Guangxi University of Science and Technology, Liuzhou 545006, China; 2 Department of Aerospace Science and Technology, Space Engineering University, Beijing 101416, China

**Keywords:** redundant rotational inertial navigation system, redundant inertial measurement unit, dual-axis rotation, fiber optic gyro, quartz accelerometer

## Abstract

A redundant rotational inertial navigation system (RRINS) comprises a redundant inertial measurement unit (RIMU) and a turntable for improving reliability and navigation accuracy. Because of the multi-sensor configuration, the RIMU has a more complex error model compared with the traditional orthogonal inertial measurement unit (IMU). Therefore, the RIMU-based rotation scheme cannot simply replicate the traditional IMU-based rotation scheme. In this study, a dual-axis rotation scheme for RIMU characteristics is proposed. First, the error model of the RIMU was established, and the error compensation of RIMU caused by rotation was analyzed. Second, the principles of rotation axis switching and reciprocating rotation were summarized, and a dual-axis rotation scheme was designed by these principles. Finally, the rotation scheme was applied to an RRINS prototype consisting of RIMU (four fiber optic gyroscopes + four quartz accelerometers) and a dual-axis turntable, and then simulations and experiments were performed. The results of the simulations and experiments show that the positioning accuracy of RRINS can be obviously improved by using the proposed rotation scheme.

## 1. Introduction

To improve the reliability of an inertial navigation system (INS), the redundant inertial measurement unit (RIMU) has been developed, and it has been widely used in aircraft, ships, land vehicles, etc. [1]. RIMU is an inertial sensing device composed of more than three accelerometers and three gyroscopes [2]. Unlike the orthogonal triaxial configuration of sensors in the traditional inertial measurement unit (IMU), the configurations of redundant sensors in RIMU are varied. These RIMU configurations can be classified as orthogonal and nonorthogonal [3]. The nonorthogonal redundant configuration is the most widely used configuration in RIMU, and includes skew redundant configuration, tetrahedron redundant configuration, dodecahedron redundant configuration, and four-cross configuration [4,5,6,7]. RIMU in navigation systems is mainly used for strapdown INS to improve the reliability of the system. In addition to improved reliability, the navigation accuracy of RIMU can also be improved by data fusion of redundant information. However, the improvement of navigation accuracy brought by data fusion is limited because of the existence of RIMU errors. Although error calibration can also improve the navigation accuracy of RIMU, RIMU error is not invariable. Therefore, compensating RIMU errors in real time is the key to improving its navigation accuracy.

With the development of inertial navigation technology, real-time error compensation technology has come into the field of view. As early as the 1980s, Levinson proposed a systematic error compensation technique based on the strapdown INS, which modulated the constant errors of IMU into periodically varying signals by regularly rotating the IMU. In the navigation calculation, the periodically varying errors will not diverge after integration, which effectively improves the navigation accuracy [8]. Subsequently, the rotational inertial navigation system (RINS) has been widely used in various areas.

In 1987, the marine ring laser inertial navigator (MARLIN), jointly developed by Sperry Marine Inc. and Honeywell Inc., greatly improved navigation accuracy by using dual-axis rotation technology [9]. In 1994, the high-precision fiber optic gyroscope inertial navigation program for strategic nuclear submarines launched by the United States proposed to develop a fiber optic gyroscope triple-axis rotational inertial navigation system [10]. In 2006, Ishibashi applied the rotation technique to the autonomous navigation of Autonomous Underwater Vehicles (AUV), and the position error was decreased to about half of the original [11]. In the early stage of RINS, rotation technology was mainly applied to high-precision inertial sensors, such as mechanical gyroscopes, ring laser gyroscopes, or fiber optic gyroscopes with mechanical or solid-state accelerometers. With the fast spread of MEMS sensors, the MEMS-based RINS have more and more applications in areas such as projectiles, land vehicles, etc. [12]. As the core of rotation technology, the rotation scheme has always been the focus of RINS research. The rotation scheme for the single-axis RINS is relatively simple. Giovanni proposed a classical 4-position single-axis rotation scheme which has been widely used [13]. However, the single-axis RINS cannot compensate for the constant errors of the inertial sensor with the input axis coinciding with the rotation axis. To compensate more constant errors, a dual-axis or triple-axis RINS is required. For the dual-axis rotation scheme, the 8-position is the simplest scheme [14]. Based on the 8-position scheme, the dual-axis rotation scheme has been extended to 16-position, 32-position, and 64-position schemes [15,16,17]. Yuan proposed a 16-position rotation scheme for dual-axis RINS, where IMU stays for 30 s after a half-cycle rotation, and the biases of the inertial sensors can be compensated after a whole period [18].

At present, most research on rotation technology is limited to traditional IMU as the research object. If RIMU is applied to RINS to form a redundant rotational inertial navigation system (RRINS), the reliability and navigation accuracy of navigation system will both be improved. There are few studies about RRINS at present. Cheng presented a dual-axis rotation method based on RIMU with a tetrahedron configuration, but the error analysis of RIMU while rotating is based on the specific redundant configuration, and it is not applicable to other redundant configurations [19]. The error analysis of the general redundant configurations for RRINS is reported in Reference [20], but its rotation scheme is a copy of the traditional IMU-based rotation scheme without any improvement for RIMU. Therefore, it is necessary to design the rotation scheme of RRINS based on the characteristics of RIMU constant errors to improve the navigation accuracy of RRINS.

In this study, a dual-axis rotation scheme for RRINS is introduced. To the best of the authors’ knowledge, the result presented in this study is the first attempt to accomplish constant error compensation by dual-axis rotation based on the RIMU error model in the RRINS.

The remainder of this paper is structured as follows. First, in Section 2, the errors of RIMU are modeled and their variations in rotation are analyzed, such as bias, scale factor error, and installation error. Second, the principles of rotation axis switching and reciprocating rotation are summarized, and a 16-point dual-axis rotation scheme is designed by these principles in Section 3. The rotation scheme was applied to an RRINS prototype, and the simulations and experiments are detailed in Section 4. Finally, Section 5 presents the conclusions of this study.

## 2. Error Model of RIMU and Error Variation in Rotation

### 2.1. General Error Model of RIMU

RIMU errors mainly consists of inertial sensor error and installation error. Inertial sensor error includes gyroscope or accelerometer bias, noise, symmetric and asymmetric scale factor error, etc. Installation error is the deviation between the actual installation direction and the design direction of the gyroscope or accelerometer. To realize the generality of the model, the RIMU error model is constructed for any sensor.

#### 2.1.1. Inertial Sensor Error Model

The inertial sensor of RIMU mainly includes a gyroscope and an accelerometer, and the sensor error is the difference between the measured value of the gyroscope or accelerometer and the real value. Taking gyroscope as an example, the relationship between the true value and the measured value of the *i*th gyroscope in RIMU can be expressed as follows:

(1) $$N_{i}^{g}=\left[1+\lambda_{i}^{g}+\mu_{i}^{g}sign\left(\omega_{i}^{g}\right)\right]\omega_{i}^{g}+b_{i}^{g}+\eta_{i}^{g}$$

where $\omega_{i}^{g}$ represents the true angular rate along with the *i*th gyroscope axis, $N_{i}^{g}$ represents the measured value of the *i*th gyroscope, and $b_{i}^{g}$, $\eta_{i}^{g}$, $\lambda_{i}^{g}$, and $\mu_{i}^{g}$ represent the bias, noise, symmetric scale factor error, and asymmetric scale factor error of the *i*th gyroscope, respectively.

Similarly, the relationship between the true value and the measured value of the *i*th accelerometer in RIMU can be expressed as follows:

(2) $$N_{i}^{a}=\left[1+\lambda_{i}^{a}+\mu_{i}^{a}sign\left(f_{i}^{a}\right)\right]f_{i}^{a}+b_{i}^{a}+\eta_{i}^{a}$$

where $f_{i}^{a}$ represents the true specific force along with the *i*th accelerometer axis, $N_{i}^{a}$ represents the measured value of the *i*th accelerometer, and $b_{i}^{a}$, $\eta_{i}^{a}$, $\lambda_{i}^{a}$, and $\mu_{i}^{a}$ represent the bias, noise, symmetric scale factor error, and asymmetric scale factor error of the *i*th accelerometer, respectively.

#### 2.1.2. Installation Error Model

**Figure 1:**
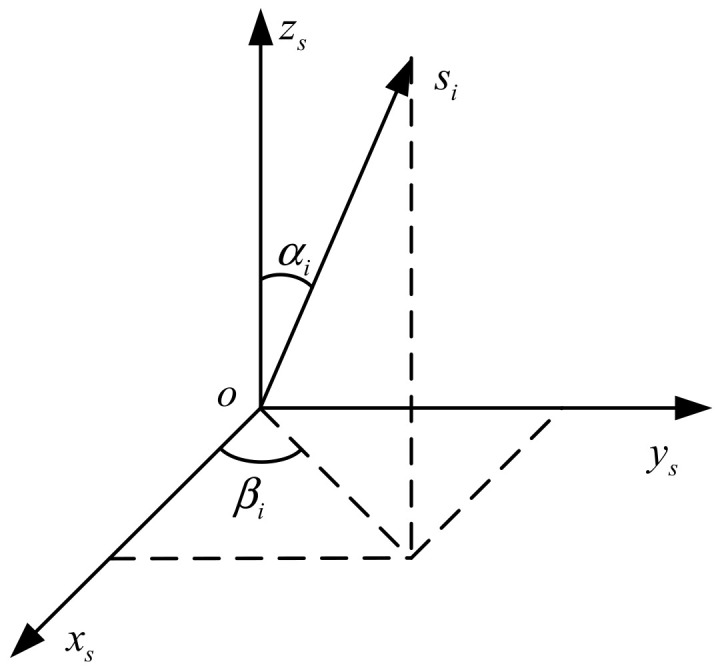
The installation direction of any single inertial sensor in RIMU. Here, $ox_{s}y_{s}z_{s}$ represents the s-frame, $s_{i}$ represents the *i*th gyroscope or accelerometer, $\alpha_{i}$ indicates the angle between the $s_{i}$ axis and the $z_{s}$ axis, and $\beta_{i}$ indicates the angle between the projection of the $s_{i}$ axis in the $ox_{s}y_{s}$ plane and the $x_{s}$ axis.

Similar to IMU, an orthogonal triaxial coordinate system (called s-frame) is built in RIMU for navigation calculation. However, the inertial sensors are not necessarily installed along the coordinate axis in RIMU. Considering the generality of the model, the installation direction of any inertial sensor in RIMU is modeled as shown in Figure 1.

Taking gyroscope as an example, without considering the installation errors, the angular rate of the *i*th gyroscope sensitive is the sum of the projection of the s-frame angular rate onto the gyroscope axis:

(3) $$\omega_{i}^{g}=\begin{array}{ccc} {}[\sin\alpha_{i}^{g}\cos\beta_{i}^{g} & \sin\alpha_{i}^{g}\sin\beta_{i}^{g} & \cos\alpha_{i}^{g}] \end{array}\left[\begin{array}{c} \omega_{x}^{s}\\ \omega_{y}^{s}\\ \omega_{z}^{s} \end{array}\right]$$

where the angular rate of the s-frame can be expressed as follows:

(4) $$\omega^{s}=\left[\begin{array}{c} \omega_{x}^{s}\\ \omega_{y}^{s}\\ \omega_{z}^{s} \end{array}\right]$$

The installation direction of the *i*th gyroscope can be expressed as the configuration vector:

(5) $$h_{i}^{g}=\begin{array}{ccc} {}[\sin\alpha_{i}^{g}\cos\beta_{i}^{g} & \sin\alpha_{i}^{g}\sin\beta_{i}^{g} & \cos\alpha_{i}^{g}] \end{array}$$

Similarly, the specific force of the *i*th accelerometer sensitive is

(6) $$f_{i}^{a}=\begin{array}{ccc} {}[\sin\alpha_{i}^{a}\cos\beta_{i}^{a} & \sin\alpha_{i}^{a}\sin\beta_{i}^{a} & \cos\alpha_{i}^{a}] \end{array}\left[\begin{array}{c} f_{x}^{s}\\ f_{y}^{s}\\ f_{z}^{s} \end{array}\right]$$

where the specific force of the s-frame can be expressed as follows:

(7) $$f^{s}=\left[\begin{array}{c} f_{x}^{s}\\ f_{y}^{s}\\ f_{z}^{s} \end{array}\right]$$

The configuration vector of the *i*th accelerometer is

(8) $$h_{i}^{a}=\begin{array}{ccc} {}[\sin\alpha_{i}^{a}\cos\beta_{i}^{a} & \sin\alpha_{i}^{a}\sin\beta_{i}^{a} & \cos\alpha_{i}^{a}] \end{array}$$

The angular rates of all gyroscopes in RIMU are expressed as a matrix:

(9) $$\omega_{}^{g}=\left[\begin{array}{c} \omega_{1}^{g}\\ \vdots \\ \omega_{m}^{g} \end{array}\right]=\left[\begin{array}{c} h_{1}^{g}\\ \vdots \\ h_{m}^{g} \end{array}\right]\left[\begin{array}{c} \omega_{x}^{s}\\ \omega_{y}^{s}\\ \omega_{z}^{s} \end{array}\right]=H^{g}\omega^{s}$$

where *m* represents the total number of gyroscopes and $H^{g}$ represents the configuration matrix of gyroscopes in RIMU.

The specific force of all accelerometers in RIMU is expressed as

(10) $$f_{}^{a}=\left[\begin{array}{c} f_{1}^{a}\\ \vdots \\ f_{n}^{a} \end{array}\right]=\left[\begin{array}{c} h_{1}^{a}\\ \vdots \\ h_{n}^{a} \end{array}\right]\left[\begin{array}{c} f_{x}^{s}\\ f_{y}^{s}\\ f_{z}^{s} \end{array}\right]==H^{a}f^{s}$$

where *n* represents the total number of accelerometers and $H^{a}$ represents the configuration matrix of accelerometers in RIMU.

**Figure 2:**
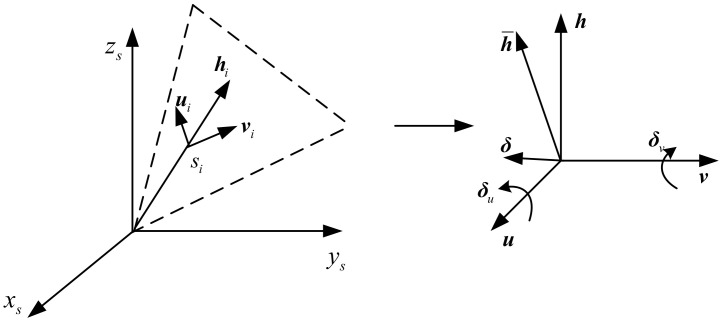
The RIMU installation error model by using rotation vector. Here, h represents the designed installation vector of the sensor (gyroscope or accelerometer), u and v are unit vectors that are perpendicular to h, $\bar{h}$ represents the actual installation vector, δ represents the rotation vector from h to $\bar{h}$, and $\delta =\delta_{u}^{}u-\delta_{v}^{}v$.

During the installation of the RIMU inertial sensor, the installation angle often has a certain deviation from the design value, which leads to the installation error of the inertial sensor, and the sensitive value is also inconsistent with the expected value. Therefore, it is necessary to establish an appropriate installation error model for correction. Considering the non-orthogonal redundancy configuration of the RIMU inertial sensors, the rotation vector is used to model the installation error as in Figure 2.

The installation error of a single inertial sensor is expressed as a rotation vector. Taking the gyroscope as an example, the designed installation vector of the *i*th gyroscope is $h_{i}^{g}$, and the actual installation vector is ${\bar{h}}_{i}^{g}$. The deviation between $h_{i}^{g}$ and ${\bar{h}}_{i}^{g}$ can be represented by a rotation vector ($\delta_{i}^{g}$):

(11) $${\bar{h}}_{i}^{g}=\left(I+\delta_{i}^{g}\times \right)h_{i}^{g}=h_{i}^{g}-h_{i}^{g}\times \delta_{i}^{g}$$

where

(12) $$\delta_{i}^{g}=\delta_{ui}^{g}u_{i}^{g}-\delta_{vi}^{g}v_{i}^{g}$$

where $h_{i}^{g}$, $u_{i}^{g}$, and $v_{i}^{g}$ are perpendicular in pairs.

Combine Equation (11) and Equation (12):

(13) $${\bar{h}}_{i}^{g}=h_{i}^{g}-\delta_{ui}^{g}v_{i}^{g}-\delta_{vi}^{g}u_{i}^{g}$$

Therefore, considering the installation errors, the angular rate of the ith gyroscope sensitive is

(14) $$\omega_{i}^{g}=\left(h_{i}^{g}-\delta_{ui}^{g}v_{i}^{g}-\delta_{vi}^{g}u_{i}^{g}\right)\omega^{s}$$

Similarly, the true specific force of the *i*th accelerometer sensitive is

(15) $$f_{i}^{a}=\left(h_{i}^{a}-\delta_{ui}^{a}v_{i}^{a}-\delta_{vi}^{a}u_{i}^{a}\right)f^{s}$$

#### 2.1.3. Vector Representation of RIMU Error Model

Combining Equation (1) and Equation (14), the measured value of the *i*th gyroscope in RIMU is

(16) $$N_{i}^{g}=\left[1+\lambda_{i}^{g}+\mu_{i}^{g}sign\left(\omega_{i}^{g}\right)\right]\left(h_{i}^{g}-\delta_{ui}^{g}v_{i}^{g}-\delta_{vi}^{g}u_{i}^{g}\right)\omega^{s}+b_{i}^{g}+\eta_{i}^{g}$$

Combining Equation (2) and Equation (15), the measured value of the *i*th accelerometer in RIMU is

(17) $$N_{i}^{a}=\left[1+\lambda_{i}^{a}+\mu_{i}^{a}sign\left(f_{i}^{a}\right)\right]\left(h_{i}^{a}-\delta_{ui}^{a}v_{i}^{a}-\delta_{vi}^{a}u_{i}^{a}\right)f^{s}+b_{i}^{a}+\eta_{i}^{a}$$

The measurements of all gyroscopes in RIMU are represented by a vector as follows:

(18) $$N^{g}=\left(I+\Lambda^{g}+{Μ}^{g}\right)\left(H^{g}-\Delta_{U}^{g}V^{g}-\Delta_{V}^{g}U^{g}\right)\omega^{s}+B^{g}+\eta^{g}$$

where

(19) $$\begin{array}{cc} \; & N^{g}={\left[\begin{array}{ccc} N_{1}^{g} & \cdots & N_{m}^{g} \end{array}\right]}^{T},H^{g}={\left[\begin{array}{ccc} H_{1}^{g} & \cdots & H_{m}^{g} \end{array}\right]}^{T}\\ \; & \Lambda^{g}=\left[\begin{array}{ccc} \lambda_{1}^{g} & \cdots & 0\\ \vdots & \ddots & \vdots \\ 0 & \cdots & \lambda_{m}^{g} \end{array}\right],M^{g}=\left[\begin{array}{ccc} \mu_{1}^{g}sign\left(\omega_{1}^{g}\right) & \cdots & 0\\ \vdots & \ddots & \vdots \\ 0 & \cdots & \mu_{m}^{g}sign\left(\omega_{m}^{g}\right) \end{array}\right]\\ \; & \Delta_{U}^{g}=\left[\begin{array}{ccc} \delta_{u1}^{g} & \cdots & 0\\ \vdots & \ddots & \vdots \\ 0 & \cdots & \delta_{um}^{g} \end{array}\right],\Delta_{V}^{g}=\left[\begin{array}{ccc} \delta_{v1}^{g} & \cdots & 0\\ \vdots & \ddots & \vdots \\ 0 & \cdots & \delta_{vm}^{g} \end{array}\right]\\ \; & V^{g}={\left[\begin{array}{ccc} v_{1}^{g} & \cdots & v_{m}^{g} \end{array}\right]}^{T},U^{g}={\left[\begin{array}{ccc} u_{1}^{g} & \cdots & u_{m}^{g} \end{array}\right]}^{T}\\ \; & B^{g}={\left[\begin{array}{ccc} b_{1}^{g} & \cdots & b_{m}^{g} \end{array}\right]}^{T},\eta^{g}={\left[\begin{array}{ccc} \eta_{1}^{g} & \cdots & \eta_{m}^{g} \end{array}\right]}^{T} \end{array}$$

Similarly, the measurements of all accelerometers in RIMU are expressed as

(20) $$N^{a}=\left(I+\Lambda^{a}+{Μ}^{a}\right)\left(H^{a}-\Delta_{U}^{a}V^{a}-\Delta_{V}^{a}U^{a}\right)f^{s}+B^{a}+\eta^{a}$$

where


(21) $$\begin{array}{cc} \; & N^{a}={\left[\begin{array}{ccc} N_{1}^{a} & \cdots & N_{n}^{a} \end{array}\right]}^{T},H^{a}={\left[\begin{array}{ccc} H_{1}^{a} & \cdots & H_{n}^{a} \end{array}\right]}^{T}\\ \; & \Lambda^{a}=\left[\begin{array}{ccc} \lambda_{1}^{a} & \cdots & 0\\ \vdots & \ddots & \vdots \\ 0 & \cdots & \lambda_{n}^{a} \end{array}\right],M^{a}=\left[\begin{array}{ccc} \mu_{1}^{a}sign\left(f_{1}^{a}\right) & \cdots & 0\\ \vdots & \ddots & \vdots \\ 0 & \cdots & \mu_{n}^{a}sign\left(f_{n}^{a}\right) \end{array}\right]\\ \; & \Delta_{U}^{a}=\left[\begin{array}{ccc} \delta_{u1}^{a} & \cdots & 0\\ \vdots & \ddots & \vdots \\ 0 & \cdots & \delta_{un}^{a} \end{array}\right],\Delta_{V}^{a}=\left[\begin{array}{ccc} \delta_{v1}^{a} & \cdots & 0\\ \vdots & \ddots & \vdots \\ 0 & \cdots & \delta_{vn}^{a} \end{array}\right]\\ \; & V^{a}={\left[\begin{array}{ccc} v_{1}^{a} & \cdots & v_{n}^{a} \end{array}\right]}^{T},U^{a}={\left[\begin{array}{ccc} u_{1}^{a} & \cdots & u_{n}^{a} \end{array}\right]}^{T}\\ \; & B^{a}={\left[\begin{array}{ccc} b_{1}^{a} & \cdots & b_{n}^{a} \end{array}\right]}^{T},\eta^{a}={\left[\begin{array}{ccc} \eta_{1}^{a} & \cdots & \eta_{n}^{a} \end{array}\right]}^{T} \end{array}$$

### 2.2. Gyroscope Error Variation in Rotation

**Figure 3:**
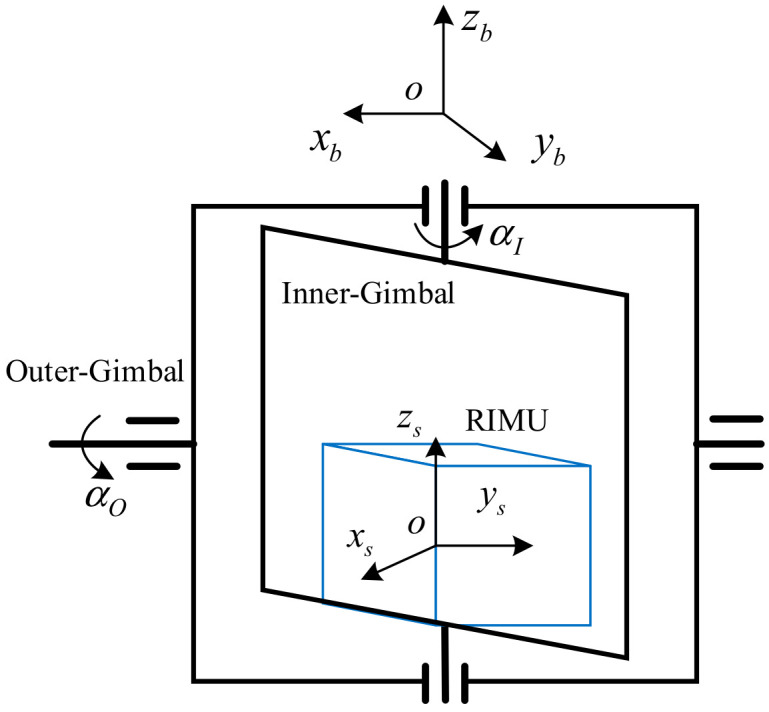
The frames in RRINS. Here, $ox_{b}y_{b}z_{b}$ represents the b-frame, $\alpha_{I}$ represents the rotation angle of the inner gimbal, and $\alpha_{O}$ represents the rotation angle of the outer gimbal.

Install the RIMU in the dual-axis turntable to build the RRINS. When RRINS rotates, there will be a deviation between the s-frame and the body frame (b-frame), as shown in Figure 3.

The conversion matrix from the s-frame to the b-frame is given as

(22) $$C_{s}^{b}=\left[\begin{array}{ccc} 1 & 0 & 0\\ 0 & \cos\alpha_{O} & -\sin\alpha_{O}\\ 0 & \sin\alpha_{O} & \cos\alpha_{O} \end{array}\right]\left[\begin{array}{ccc} \cos\alpha_{I} & -\sin\alpha_{I} & 0\\ \sin\alpha_{I} & \cos\alpha_{I} & 0\\ 0 & 0 & 1 \end{array}\right]$$

According to Figure 1, the projection of a single gyroscope measurement into the s-frame is as follows:

(23) $$\omega_{i}^{s}=\left[\begin{array}{c} \omega_{xi}^{s}\\ \omega_{yi}^{s}\\ \omega_{zi}^{s} \end{array}\right]=\left[\begin{array}{c} N_{i}^{g}\sin\alpha_{i}\cos\beta_{i}\\ N_{i}^{g}\sin\alpha_{i}\sin\beta_{i}\\ N_{i}^{g}\cos\alpha_{i} \end{array}\right]$$

As RIMU rotates, the projection of a single gyroscope measurement into the b-frame is as follows:

(24) $$\omega_{i}^{b}=\left[\begin{array}{c} \omega_{xi}^{b}\\ \omega_{yi}^{b}\\ \omega_{zi}^{b} \end{array}\right]=C_{s}^{b}\left[\begin{array}{c} N_{i}^{g}\sin\alpha_{i}\cos\beta_{i}\\ N_{i}^{g}\sin\alpha_{i}\sin\beta_{i}\\ N_{i}^{g}\cos\alpha_{i} \end{array}\right]$$

Expand Equation (16), ignoring the higher-order minor terms, considering only the constant errors, and the gyroscope measurement value can be obtained as

(25) $$N_{i}^{g}=h_{i}^{g}\omega^{s}+\lambda_{i}^{g}h_{i}^{g}\omega^{s}+\mu_{i}^{g}sign\left(\omega_{i}^{g}\right)h_{i}^{g}\omega^{s}-\left(\delta_{ui}^{g}v_{i}^{g}+\delta_{vi}^{g}u_{i}^{g}\right)\omega^{s}+b_{i}^{g}$$

where $\lambda_{i}^{g}h_{i}^{g}\omega^{s}$ is the measurement error caused by symmetric scale factor error (MESSF), $\mu_{i}^{g}sign\left(\omega_{i}^{g}\right)h_{i}^{g}\omega^{s}$ is the measurement error caused by asymmetric scale factor error (MEASF), and $\left(\delta_{ui}^{g}v_{i}^{g}+\delta_{vi}^{g}u_{i}^{g}\right)\omega^{s}$ is the measurement error caused by installation error (MEIE).

It can be seen that the factors affecting the measurement accuracy of the gyroscope are MESSF, MEASF, MEIE, and bias. As RIMU rotates, the projection of these errors into the b-frame will change.

#### 2.2.1. Gyroscope Bias Variation

When RIMU is rotated $\alpha_{I}$ around the $z_{s}$ axis, and $\alpha_{O}=0$, the projection of the gyroscope bias into the b-frame is

(26) $$\left[\begin{array}{c} \varepsilon_{xi}^{b}\\ \varepsilon_{yi}^{b}\\ \varepsilon_{zi}^{b} \end{array}\right]=\left[\begin{array}{c} b_{i}^{g}\sin\alpha_{i}\cos\beta_{i}\cos\alpha_{I}-b_{i}^{g}\sin\alpha_{i}\sin\beta_{i}\sin\alpha_{I}\\ b_{i}^{g}\sin\alpha_{i}\cos\beta_{i}\sin\alpha_{I}+b_{i}^{g}\sin\alpha_{i}\sin\beta_{i}\cos\alpha_{I}\\ b_{i}^{g}\cos\alpha_{i} \end{array}\right]$$

When RIMU is rotated $\alpha_{O}$ around the $x_{s}$ axis, and $\alpha_{I}=0$, the projection of the gyroscope bias into the b-frame is

(27) $$\left[\begin{array}{c} \varepsilon_{xi}^{b}\\ \varepsilon_{yi}^{b}\\ \varepsilon_{zi}^{b} \end{array}\right]=\left[\begin{array}{c} b_{i}^{g}\sin\alpha_{i}\cos\beta_{i}\\ b_{i}^{g}\sin\alpha_{i}\sin\beta_{i}\cos\alpha_{O}+b_{i}^{g}\cos\alpha_{i}\sin\alpha_{O}\\ -b_{i}^{g}\sin\alpha_{i}\sin\beta_{i}\sin\alpha_{O}+b_{i}^{g}\cos\alpha_{i}\cos\alpha_{O} \end{array}\right]$$

It can be seen from Equations (26) and (27) that if RIMU is rotated periodically around one axis, the bias projected by the gyroscope on the other two axes can be completely compensated. If the two axes are rotated alternately, the bias of a single gyroscope can be completely compensated. Since the gyroscope model is a general model, the bias of all gyroscopes in RIMU can be compensated by the dual-axis rotation.

#### 2.2.2. Gyroscope MESSF Variation

The projection of gyroscope MESSF into the b-frame is

(28) $$\delta \omega_{\lambda i}^{b}=C_{s}^{b}\lambda_{i}^{g}{}_{i}h_{i}^{gT}h_{i}^{g}\omega^{s}$$

Because the rotational angular rate of the turntable is much higher than that of the Earth, the influence of the Earth’s rotational angular rate can be ignored. Thus, when RRINS is stationary, the angular rate of RIMU is derived only from the rotational angular rate of the turntable.

When RIMU is rotated $\alpha_{I}$ around the $z_{s}$ axis, and $\alpha_{O}=0$, the projection of the gyroscope MESSF into the b-frame is

(29) $$\begin{array}{l} \delta \omega_{\lambda i}^{b}=C_{s}^{b}\lambda_{i}^{g}h_{i}^{gT}h_{i}^{g}\left[\begin{array}{c} 0\\ 0\\ {\dot{\alpha }}_{I} \end{array}\right]\\ =\lambda_{i}^{g}{\dot{\alpha }}_{I}\cos\alpha_{i}\left[\begin{array}{c} \cos\alpha_{I}\sin\alpha_{i}\cos\beta_{i}-\sin\alpha_{I}\sin\alpha_{i}\sin\beta_{i}\\ \sin\alpha_{I}\sin\alpha_{i}\cos\beta_{i}+\cos\alpha_{I}\sin\alpha_{i}\sin\beta_{i}\\ \cos\alpha_{i} \end{array}\right] \end{array}$$

When RIMU is rotated $\alpha_{O}$ around the $x_{s}$ axis, and $\alpha_{I}=0$, the projection of the gyroscope MESSF into the b-frame is

(30) $$\begin{array}{l} \delta \omega_{\lambda i}^{b}=C_{s}^{b}\lambda_{i}^{g}h_{i}^{gT}h_{i}^{g}\left[\begin{array}{c} {\dot{\alpha }}_{O}\\ 0\\ 0 \end{array}\right]\\ =\lambda_{i}^{g}{\dot{\alpha }}_{O}\sin\alpha_{i}\cos\beta_{i}\left[\begin{array}{c} \sin\alpha_{i}\cos\beta_{i}\\ \cos\alpha_{O}\sin\alpha_{i}\sin\beta_{i}-\sin\alpha_{O}\cos\alpha_{i}\\ \sin\alpha_{O}\sin\alpha_{i}\sin\beta_{i}+\cos\alpha_{O}\cos\alpha_{i} \end{array}\right] \end{array}$$

$\lambda_{i}^{g}$, $\alpha_{i}$, and $\beta_{i}$ are all constants. When the axis of rotation rotates at a constant speed, ${\dot{\alpha }}_{I}$ and ${\dot{\alpha }}_{O}$ are also constants. Therefore, the MESSF of the other two non-rotational axes can be completely compensated after one cycle of rotation. If the rotation is positive and negative in the two rotation cycles, respectively, the MESSF of the rotation axis can be completely compensated. In conclusion, the gyroscope MESSF can be completely compensated by single-axis reciprocating rotation or dual-axis rotation.

#### 2.2.3. Gyroscope MEASF Variation

The projection of gyroscope MEASF into the b-frame is

(31) $$\delta \omega_{\mu i}^{b}=C_{s}^{b}\mu_{i}^{g}sign\left(h_{i}^{g}\omega^{s}\right)h_{i}^{gT}h_{i}^{g}\omega^{s}$$

When RIMU is rotated $\alpha_{I}$ around the $z_{s}$ axis, and $\alpha_{O}=0$, the projection of the gyroscope MEASF into the b-frame is

(32) $$\begin{array}{l} \delta \omega_{\mu i}^{b}=C_{s}^{b}\mu_{i}^{g}sign\left({\dot{\alpha }}_{I}\cos\alpha_{i}\right)h_{i}^{gT}h_{i}^{g}\left[\begin{array}{c} 0\\ 0\\ {\dot{\alpha }}_{I} \end{array}\right]\\ =\mu_{i}^{g}sign\left({\dot{\alpha }}_{I}\cos\alpha_{i}\right){\dot{\alpha }}_{I}\cos\alpha_{i}\left[\begin{array}{c} \cos\alpha_{I}\sin\alpha_{i}\cos\beta_{i}-\sin\alpha_{I}\sin\alpha_{i}\sin\beta_{i}\\ \sin\alpha_{I}\sin\alpha_{i}\cos\beta_{i}+\cos\alpha_{I}\sin\alpha_{i}\sin\beta_{i}\\ \cos\alpha_{i} \end{array}\right] \end{array}$$

When RIMU is rotated $\alpha_{O}$ around the $x_{s}$ axis, and $\alpha_{I}=0$, the projection of the gyroscope MEASF into the b-frame is

(33) $$\begin{array}{l} \delta \omega_{\mu i}^{b}=C_{s}^{b}\mu_{i}^{g}sign\left({\dot{\alpha }}_{O}\sin\alpha_{i}\cos\beta_{i}\right)h_{i}^{gT}h_{i}^{g}\left[\begin{array}{c} {\dot{\alpha }}_{O}\\ 0\\ 0 \end{array}\right]\\ =\mu_{i}^{g}sign\left({\dot{\alpha }}_{O}\sin\alpha_{i}\cos\beta_{i}\right){\dot{\alpha }}_{O}\sin\alpha_{i}\cos\beta_{i}\left[\begin{array}{c} \sin\alpha_{i}\cos\beta_{i}\\ \cos\alpha_{O}\sin\alpha_{i}\sin\beta_{i}-\sin\alpha_{O}\cos\alpha_{i}\\ \sin\alpha_{O}\sin\alpha_{i}\sin\beta_{i}+\cos\alpha_{O}\cos\alpha_{i} \end{array}\right] \end{array}$$

Similar to MESSF, the gyroscope MEASF of the other two non-rotational axes can be completely compensated after one cycle of rotation. Because of the anti-symmetry of the asymmetric scaling factor error, the MEASF continues to diverge in both positive and negative rotation. Therefore, dual-axis rotation is required to compensate the gyroscope MEASF completely.

#### 2.2.4. Gyroscope MEIE Variation

The projection of gyroscope MEIE into the b-frame is

(34) $$\delta \omega_{uvi}^{b}=-C_{s}^{b}h_{i}^{gT}\left(\delta_{ui}^{g}v_{i}^{g}+\delta_{vi}^{g}u_{i}^{g}\right)\omega^{s}$$

Since $h_{i}^{gT}\left(\delta_{ui}^{g}v_{i}^{g}+\delta_{vi}^{g}u_{i}^{g}\right)$ is a constant matrix, the variation in the gyroscope MEIE is the same as that of the gyroscope MESSF. The gyroscope MEIE can be completely compensated by single-axis reciprocating rotation or dual-axis rotation.

### 2.3. Accelerometer Error Variation in Rotation

Expand Equation (17), ignoring the higher-order minor terms, considering only the constant errors, and the accelerometer measurement value can be obtained as

(35) $$N_{j}^{a}=h_{j}^{a}f^{s}+\lambda_{j}^{a}h_{j}^{a}f^{s}+\mu_{j}^{a}sign\left(f_{j}^{a}\right)h_{j}^{a}f^{s}-\left(\delta_{uj}^{a}v_{j}^{a}+\delta_{vj}^{a}u_{j}^{a}\right)f^{s}+b_{j}^{a}$$

The factors affecting the measurement accuracy of accelerometer are MESSF, MEASF, MEIE, and bias. As RIMU rotates, the projection of these errors into the b-frame will change.

#### 2.3.1. Accelerometer Bias Variation

The bias variation in the accelerometer is the same as that of the gyroscope when RIMU rotates. Therefore, the bias of all accelerometers in RIMU can be compensated by the dual-axis rotation.

#### 2.3.2. Accelerometer MESSF Variation

When RRINS is stationary, the specific force of RIMU is derived only from the Earth’s gravity. Therefore, the projection of the accelerometer MESSF into the b-frame is

(36) $$\delta f_{\lambda j}^{b}=C_{s}^{b}\delta f_{\lambda j}^{s}=\lambda_{j}^{a}C_{s}^{b}h_{j}^{aT}h_{j}^{a}C_{b}^{s}\left[\begin{array}{c} 0\\ 0\\ G \end{array}\right]$$

When RIMU is rotated $\alpha_{I}$ around the $z_{s}$ axis, and $\alpha_{O}=0$, Equation (36) can be expanded as

(37) $$\delta f_{\lambda j}^{b}=\lambda_{j}^{a}G\cos\alpha_{j}\left[\begin{array}{c} \cos\alpha_{I}\sin\alpha_{j}\cos\beta_{j}-\sin\alpha_{I}\sin\alpha_{j}\sin\beta_{j}\\ \sin\alpha_{I}\sin\alpha_{j}\cos\beta_{j}+\cos\alpha_{I}\sin\alpha_{j}\sin\beta_{j}\\ \cos\alpha_{j} \end{array}\right]$$

When RIMU is rotated $\alpha_{O}$ around the $x_{s}$ axis, and $\alpha_{I}=0$, Equation (36) can be expanded as

(38) $$\begin{array}{l} \delta f_{\lambda j}^{b}=\\ \lambda_{j}^{a}G\left[\begin{array}{c} \sin\alpha_{O}\sin^{2}\alpha_{j}\sin\beta_{j}\cos\beta_{j}+\cos\alpha_{O}\sin\alpha_{j}\cos\alpha_{j}\cos\beta_{j}\\ \frac{1}{2}\sin2\alpha_{O}\left(\sin^{2}\alpha_{j}\sin^{2}\beta_{j}-\cos^{2}\alpha_{j}\right)+\cos2\alpha_{O}\sin\alpha_{j}\cos\alpha_{j}\sin\beta_{j}\\ \sin^{2}\alpha_{O}\sin^{2}\alpha_{j}\sin^{2}\beta_{j}+\cos^{2}\alpha_{O}\cos^{2}\alpha_{j}+\sin2\alpha_{O}\sin\alpha_{j}\cos\alpha_{j}\sin\beta_{j} \end{array}\right] \end{array}$$

Because of the presence of $\sin^{2}\alpha_{O}$ and $\cos^{2}\alpha_{O}$, the MESSF in the $z_{b}$ axis is always not negative. Therefore, no matter how RIMU rotates, the projection of MESSF on $z_{b}$ cannot be compensated, but the projection of MESSF on $x_{b}$ and $y_{b}$ can be compensated.

#### 2.3.3. Accelerometer MEASF Variation

The projection of the accelerometer MEASF into the b-frame is

(39) $$\delta f_{\mu j}^{b}=C_{s}^{b}\delta f_{\mu j}^{s}=\mu_{j}^{a}sign\left(f_{j}^{a}\right)C_{s}^{b}h_{j}^{aT}h_{j}^{a}C_{b}^{s}\left[\begin{array}{c} 0\\ 0\\ G \end{array}\right]$$

When RIMU is rotated $\alpha_{I}$ around the $z_{s}$ axis, and $\alpha_{O}=0$, Equation (39) can be expanded as

(40) $$\begin{array}{l} \delta f_{\mu j}^{b}=\mu_{j}^{a}sign\left(\cos\alpha_{j}\right)G\cos\alpha_{j}\\ \left[\begin{array}{c} \cos\alpha_{I}\sin\alpha_{j}\cos\beta_{j}-\sin\alpha_{I}\sin\alpha_{j}\sin\beta_{j}\\ \sin\alpha_{I}\sin\alpha_{j}\cos\beta_{j}+\cos\alpha_{I}\sin\alpha_{j}\sin\beta_{j}\\ \cos\alpha_{j} \end{array}\right] \end{array}$$

When RIMU is rotated $\alpha_{O}$ around the $x_{s}$ axis, and $\alpha_{I}=0$, Equation (39) can be expanded as

(41) $$\begin{array}{l} \delta f_{\mu j}^{b}=\mu_{j}^{a}Gsign\left(\sin\alpha_{O}\sin\alpha_{j}\sin\beta_{j}+\cos\alpha_{O}\cos\alpha_{j}\right)\\ \left[\begin{array}{c} \sin\alpha_{j}\cos\beta_{j}\left(\sin\alpha_{O}\sin\alpha_{j}\sin\beta_{j}+\cos\alpha_{O}\cos\alpha_{j}\right)\\ \left(\sin\alpha_{O}\sin\alpha_{j}\sin\beta_{j}+\cos\alpha_{O}\cos\alpha_{j}\right)\left(\cos\alpha_{O}\sin\alpha_{j}\sin\beta_{j}-\sin\alpha_{O}\cos\alpha_{j}\right)\\ {\left(\sin\alpha_{O}\sin\alpha_{j}\sin\beta_{j}+\cos\alpha_{O}\cos\alpha_{j}\right)}^{2} \end{array}\right] \end{array}$$

It can be seen that the accelerometer MEASF can be compensated on the $x_{b}$ and $y_{b}$ axes when the $z_{s}$ axis rotates at a constant speed. The accelerometer MEASF can be compensated on the $y_{b}$ and $z_{b}$ axes when the $x_{s}$ axis rotates at a constant speed. Therefore, the accelerometer MEASF can be completely compensated by the dual-axis rotation.

#### 2.3.4. Accelerometer MEIE Variation

The variation in the accelerometer MEIE is the same as that of the accelerometer MESSF. Therefore, no matter how RIMU rotates, the projection of MEIE on $z_{b}$ cannot be compensated.

## 3. Dual-Axis Rotation Scheme

It can be seen from Section 2 that the dual-axis rotation can compensate all the constant errors of the gyroscopes and most of the constant errors of the accelerometers; thus, the RRINS navigation accuracy can be improved. However, there are some problems in dual-axis rotation, such as how to switch the rotation axis and how to reverse the reciprocating rotation. Therefore, it is necessary to summarize the principle of rotation axis switching and reciprocating rotation to provide a basis for the design of the rotation scheme.

### 3.1. Rotation Axis Switching Principle

In dual-axis rotation, if two axes rotate at the same time, the rotation motion of RIMU will be complicated and its compensation effect will be destroyed. Therefore, a time-sharing rotation scheme is generally adopted in dual-axis rotation; that is, there can only be one axis rotation at a time. When to switch the rotation axis in a dual-axis time-sharing rotation is a problem that needs to be solved.

Taking the bias of gyroscope in RIMU as an example, assuming that all rotation axes rotate at a constant angular rate Ω, when one axis rotates for a cycle before the rotation of the other axis, the error integral in b-frame is

(42) $$\left\{\begin{array}{cc} \; & \int_{0}^{2T}\varepsilon_{xi}^{b}dt=\int_{0}^{T}\left(b_{i}^{g}\sin\alpha_{i}\cos\beta_{i}\cos\Omega t-b_{i}^{g}\sin\alpha_{i}\sin\beta_{i}\sin\Omega t\right)dt+\int_{T}^{2T}b_{i}^{g}\sin\alpha_{i}\cos\beta_{i}dt\\ \; & \int_{0}^{2T}\varepsilon_{yi}^{b}dt=\int_{0}^{T}\left(b_{i}^{g}\sin\alpha_{i}\cos\beta_{i}\sin\Omega t+b_{i}^{g}\sin\alpha_{i}\sin\beta_{i}\cos\Omega t\right)dt\\ \; & +\int_{T}^{2T}\left(b_{i}^{g}\sin\alpha_{i}\sin\beta_{i}\cos\Omega t+b_{i}^{g}\cos\alpha_{i}\sin\Omega t\right)dt\\ \; & \int_{0}^{2T}\varepsilon_{zi}^{b}dt=\int_{0}^{T}b_{i}^{g}\cos\alpha_{i}dt+\int_{T}^{2T}\left(-b_{i}^{g}\sin\alpha_{i}\sin\beta_{i}\sin\Omega t+b_{i}^{g}\cos\alpha_{i}\cos\Omega t\right)dt \end{array}\right.$$

where *T* represents the cycle of a single rotation of 360°.

If the rotation axis is switched after the full-cycle rotation of an axis, the errors along the rotation axis during the rotation will be accumulated and cannot be compensated. With dual-axis rotation in this way, only the error in $y_{b}$ is fully compensated. The direction of the rotation axis in the b-frame should be changed to solve the error accumulation problem of the rotation axis. To reverse the rotation axis, the rotation axis cannot be switched after a full-cycle rotation of an axis, but after a half-cycle rotation (180°).

The rotation of two full cycles is divided into four half-cycle rotations. For the convenience of analysis, the rotation of four stages is called 4-position, and the single stage is called one position. The rotation angle of the rotation axis in one position is 180°. The four stages of rotation are as follows.

Position 1: RIMU is rotated around $z_{s}$, $\alpha_{O}=0$, the conversion matrix from the s-frame to the b-frame is

(43) $$C_{s1}^{b}=\left[\begin{array}{ccc} \cos\alpha_{I} & -\sin\alpha_{I} & 0\\ \sin\alpha_{I} & \cos\alpha_{I} & 0\\ 0 & 0 & 1 \end{array}\right]$$

Position 2: RIMU is rotated around $x_{s}$, $\alpha_{I}=180$, the conversion matrix from the s-frame to the b-frame is

(44) $$C_{s2}^{b}=\left[\begin{array}{ccc} -1 & 0 & 0\\ 0 & -\cos\alpha_{O} & -\sin\alpha_{O}\\ 0 & -\sin\alpha_{O} & \cos\alpha_{O} \end{array}\right]$$

Position 3: RIMU is rotated around $z_{s}$, $\alpha_{O}=180$, the conversion matrix from the s-frame to the b-frame is

(45) $$C_{s3}^{b}=\left[\begin{array}{ccc} \cos\alpha_{I} & -\sin\alpha_{I} & 0\\ -\sin\alpha_{I} & -\cos\alpha_{I} & 0\\ 0 & 0 & -1 \end{array}\right]$$

Position 4: RIMU is rotated around $x_{s}$, $\alpha_{I}=0$, the conversion matrix from the s-frame to the b-frame is

(46) $$C_{s4}^{b}=\left[\begin{array}{ccc} 1 & 0 & 0\\ 0 & \cos\alpha_{O} & -\sin\alpha_{O}\\ 0 & \sin\alpha_{O} & \cos\alpha_{O} \end{array}\right]$$

Since a position rotates only 180°, the rotation axis of RIMU reverses, and the projection of the uncompensated constant error of this axis in the s-frame reverses. Therefore, if the period of each position is the same, the rotation axis error after the completion of the 4-position can be compensated.

According to the above analysis, the minimum rotation unit of dual-axis rotation is specified as the one position, which refers to the rotation of 180°. The principle of the rotation axis switching in dual-axis rotation is that the rotation axis should be switched immediately after the one position (180°).

### 3.2. Reciprocating Rotation Principle

It can be seen from Section 2 that reciprocating rotation can compensate for more errors than unidirectional rotation. Therefore, reciprocating rotation of an axis according to the principle of symmetry is necessary. According to the principle of rotation axis switching, there are two ways to switch the direction of the rotation axis: reverse after a full-cycle rotation (360°) or a half-cycle rotation (180°) for the same rotation axis.

Taking the bias of gyroscope in RIMU 4-position rotation as an example, in the case of reversing rotation after a full-cycle rotation, the error integral in $x_{b}$ is

(47) $$\begin{array}{l} \int_{0}^{2T}\varepsilon_{xi}^{b}dt=\int_{0}^{T/2}\left(b_{i}^{g}\sin\alpha_{i}\cos\beta_{i}\cos\Omega t-b_{i}^{g}\sin\alpha_{i}\sin\beta_{i}\sin\Omega t\right)dt\\ +\int_{0}^{T/2}\left(-b_{i}^{g}\sin\alpha_{i}\cos\beta_{i}\right)dt\\ +\int_{0}^{T/2}\left[b_{i}^{g}\sin\alpha_{i}\cos\beta_{i}\cos\left(\pi -\Omega t\right)-b_{i}^{g}\sin\alpha_{i}\sin\beta_{i}\sin\left(\pi -\Omega t\right)\right]dt\\ +\int_{0}^{T/2}\left(b_{i}^{g}\sin\alpha_{i}\cos\beta_{i}\right)dt \end{array}$$

The error integrals of $b_{i}^{g}\sin\alpha_{i}\sin\beta_{i}\sin\Omega t$ and $b_{i}^{g}\sin\alpha_{i}\sin\beta_{i}\sin\left(\pi -\Omega t\right)$ are not zeros after rotating a 4-position. Reverse rotation is required in the next 4-position to fully compensate the error in $x_{b}$.

The error integral in $y_{b}$ is

(48) $$\begin{array}{l} \int_{0}^{2T}\varepsilon_{yi}^{b}dt=\int_{0}^{T/2}\left(b_{i}^{g}\sin\alpha_{i}\cos\beta_{i}\sin\Omega t+b_{i}^{g}\sin\alpha_{i}\sin\beta_{i}\cos\Omega t\right)dt\\ +\int_{0}^{T/2}\left(-b_{i}^{g}\sin\alpha_{i}\sin\beta_{i}\cos\Omega t-b_{i}^{g}\cos\alpha_{i}\sin\Omega t\right)dt\\ +\int_{0}^{T/2}\left[-b_{i}^{g}\sin\alpha_{i}\cos\beta_{i}\sin\left(\pi -\Omega t\right)-b_{i}^{g}\sin\alpha_{i}\sin\beta_{i}\cos\left(\pi -\Omega t\right)\right]dt\\ +\int_{0}^{T/2}\left[b_{i}^{g}\sin\alpha_{i}\sin\beta_{i}\cos\left(\pi +\Omega t\right)-b_{i}^{g}\cos\alpha_{i}\sin\left(\pi +\Omega t\right)\right]dt \end{array}$$

The error in $y_{b}$ can be completely compensated after a 4-position rotation.

The error integral in $z_{b}$ is

(49) $$\begin{array}{l} \int_{0}^{2T}\varepsilon_{zi}^{b}dt=\int_{0}^{T/2}\left(b_{i}^{g}\cos\alpha_{i}\right)dt\\ +\int_{0}^{T/2}\left(-b_{i}^{g}\sin\alpha_{i}\sin\beta_{i}\sin\Omega t+b_{i}^{g}\cos\alpha_{i}\cos\Omega t\right)dt\\ +\int_{0}^{T/2}\left(-b_{i}^{g}\cos\alpha_{i}\right)dt\\ +\int_{0}^{T/2}\left[b_{i}^{g}\sin\alpha_{i}\sin\beta_{i}\sin\left(\pi +\Omega t\right)+b_{i}^{g}\cos\alpha_{i}\cos\left(\pi +\Omega t\right)\right]dt \end{array}$$

The error integrals of $b_{i}^{g}\sin\alpha_{i}\sin\beta_{i}\sin\Omega t$ and $b_{i}^{g}\sin\alpha_{i}\sin\beta_{i}\sin\left(\pi +\Omega t\right)$ are not zeros after rotating a 4-position. Reverse rotation is required in the next 4-position to fully compensate the error in $z_{b}$.

Therefore, in the case of reversing rotation after a half-cycle rotation, the error compensation in $y_{b}$ only requires a 4-position, whereas the error compensation in $x_{b}$ and $z_{b}$ requires two 4-positions.

In the case of reversing rotation after a half-cycle rotation, the error integral in $x_{b}$ is

(50) $$\begin{array}{l} \int_{0}^{2T}\varepsilon_{xi}^{b}dt=\int_{0}^{T/2}\left(b_{i}^{g}\sin\alpha_{i}\cos\beta_{i}\cos\Omega t-b_{i}^{g}\sin\alpha_{i}\sin\beta_{i}\sin\Omega t\right)dt\\ +\int_{0}^{T/2}\left(-b_{i}^{g}\sin\alpha_{i}\cos\beta_{i}\right)dt\\ +\int_{0}^{T/2}\left[b_{i}^{g}\sin\alpha_{i}\cos\beta_{i}\cos\left(\pi +\Omega t\right)-b_{i}^{g}\sin\alpha_{i}\sin\beta_{i}\sin\left(\pi +\Omega t\right)\right]dt\\ +\int_{0}^{T/2}\left(b_{i}^{g}\sin\alpha_{i}\cos\beta_{i}\right)dt \end{array}$$

The error in $x_{b}$ can be completely compensated after a 4-position rotation.

The error integral in $y_{b}$ is

(51) $$\begin{array}{l} \int_{0}^{2T}\varepsilon_{yi}^{b}dt=\int_{0}^{T/2}\left(b_{i}^{g}\sin\alpha_{i}\cos\beta_{i}\sin\Omega t+b_{i}^{g}\sin\alpha_{i}\sin\beta_{i}\cos\Omega t\right)dt\\ +\int_{0}^{T/2}\left(-b_{i}^{g}\sin\alpha_{i}\sin\beta_{i}\cos\Omega t-b_{i}^{g}\cos\alpha_{i}\sin\Omega t\right)dt\\ +\int_{0}^{T/2}\left[-b_{i}^{g}\sin\alpha_{i}\cos\beta_{i}\sin\left(\pi +\Omega t\right)-b_{i}^{g}\sin\alpha_{i}\sin\beta_{i}\cos\left(\pi +\Omega t\right)\right]dt\\ +\int_{0}^{T/2}\left[b_{i}^{g}\sin\alpha_{i}\sin\beta_{i}\cos\left(\pi -\Omega t\right)-b_{i}^{g}\cos\alpha_{i}\sin\left(\pi -\Omega t\right)\right]dt \end{array}$$

The error integrals of $b_{i}^{g}\sin\alpha_{i}\cos\beta_{i}\sin\Omega t$ and $b_{i}^{g}\sin\alpha_{i}\cos\beta_{i}\sin\left(\pi +\Omega t\right)$ are not zeros after rotating a 4-position. Reverse rotation is required in the next 4-position to fully compensate the error in $y_{b}$.

The error integral in $z_{b}$ is

(52) $$\begin{array}{l} \int_{0}^{2T}\varepsilon_{zi}^{b}dt=\int_{0}^{T/2}\left(b_{i}^{g}\cos\alpha_{i}\right)dt\\ +\int_{0}^{T/2}\left(-b_{i}^{g}\sin\alpha_{i}\sin\beta_{i}\sin\Omega t+b_{i}^{g}\cos\alpha_{i}\cos\Omega t\right)dt\\ +\int_{0}^{T/2}\left(-b_{i}^{g}\cos\alpha_{i}\right)dt\\ +\int_{0}^{T/2}\left[b_{i}^{g}\sin\alpha_{i}\sin\beta_{i}\sin\left(\pi -\Omega t\right)+b_{i}^{g}\cos\alpha_{i}\cos\left(\pi -\Omega t\right)\right]dt \end{array}$$

The error in $z_{b}$ can be completely compensated after a 4-position rotation.

Therefore, in the case of reversing rotation after a half-cycle rotation, the error compensation in $x_{b}$ and $z_{b}$ only requires a 4-position, whereas the error compensation in $y_{b}$ requires two 4-positions.

The error in $x_{b}$ and $z_{b}$ diverge longer using the method of rotation after a full-cycle rotation, while $y_{b}$ diverges longer using the method of reversing rotation after a half-cycle rotation. In the global consideration, the reciprocating rotation principle is reversing rotation after a half-cycle rotation.

### 3.3. Rotation Scheme Design

**Figure 4:**
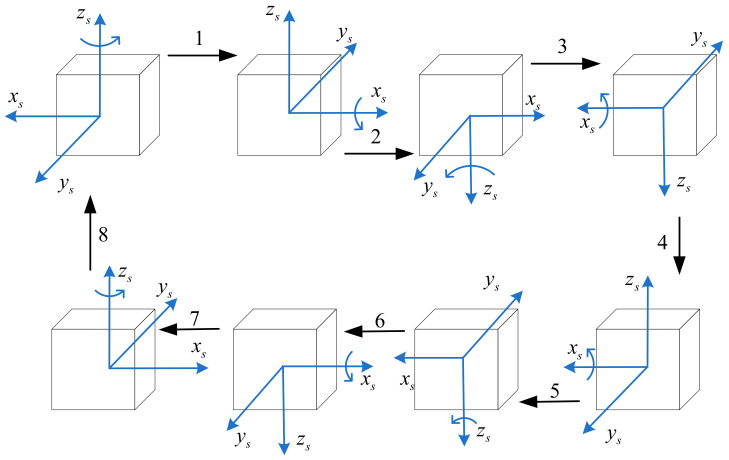
The 8-position dual-axis rotation scheme. Here, 1, 2, …, and 8 represent the 1st, 2nd, … , and 8th rotation order respectively.

According to the rotation axis switching principle and the reciprocating rotation principle, and considering the characteristics of RIMU errors, a 4-position dual-axis rotation is constructed. In 4-position, the two axes rotate alternately and rotate in reverse after half a cycle. The rotation axis switching after rotating 180° can make the accumulated errors along the rotation axis cancel each other out according to the rotation axis switching principle. The rotation axis rotates in reverse after rotating 180° and can compensate more accumulated axial errors in a shorter time according to the reciprocating rotation principle. However, the full-cycle rotation is not completed in a single direction, and the errors cannot be completely compensated. Therefore, one more 4-position needs to be added to the first 4-position. The second 4-position is the reverse process of the first 4-position, and the two 4-positions constitute an 8-position, which realizes a full-cycle rotation in each direction, and thus completely compensates the RIMU errors. The 8-position dual-axis rotation scheme is shown in Figure 4.

The 8-position can compensate for the RIMU errors to the greatest extent in theory. However, the rotation axis control error in the actual system will lead to uneven rotation speed. In addition, the rotation axis of the inner frame always rotates in the same direction in the 8-positon, which will lead to the accumulation of errors and mechanical wear. Therefore, one more 8-position needs to be added to the first 8-position; according to the principle of symmetry, the two 8-position constitute a 16-position, as shown in Table 1.

After RRINS are rotated according to the 16-position rotation scheme, the projection of the RIMU constant errors in b-frame will become periodic. In the calculation of inertial navigation, the constant errors of RIMU will be integrated. These periodical constant errors will be cancelled out after the integration of a 16-position period, so as to realize the real-time compensation of the constant errors in RIMU.

## 4. RRINS Prototype and Experiment

### 4.1. RRINS Prototype

**Figure 5:**
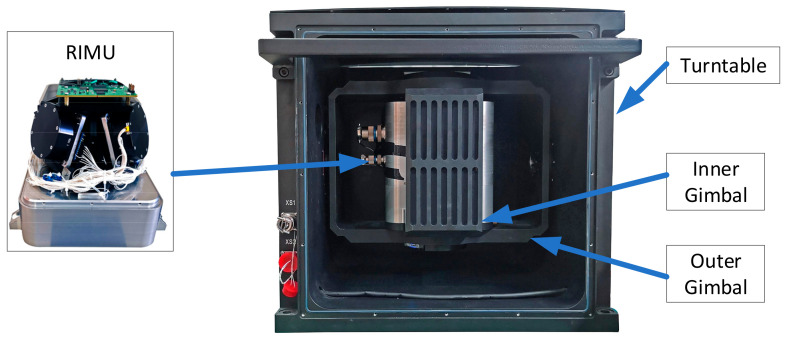
RRINS prototype.

As is shown in Figure 5, we designed a RRINS prototype consisting of a tetrahedron RIMU and a dual-axis turntable.

**Figure 6:**
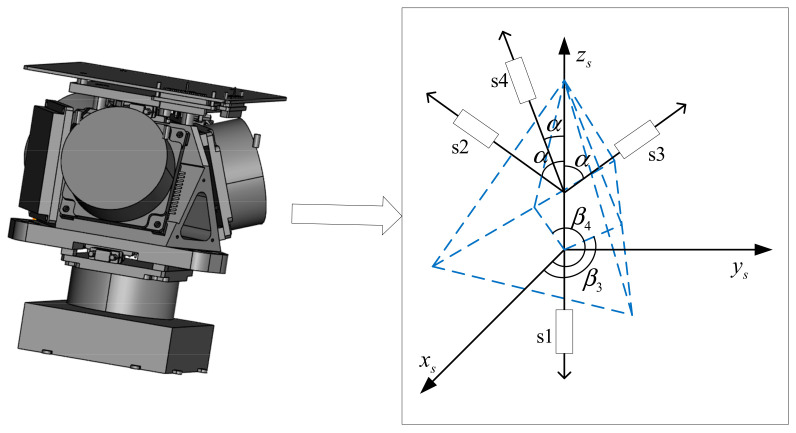
Configuration structure of RIMU. Here, *si* indicates the *i*th group sensors (*i* = 1,2,3,4). The group sensors include a gyroscope and accelerometer.

The RIMU in RRINS consisted of four fiber optic gyroscopes and four quartz accelerometers, and the parameters of gyroscopes and accelerometers are shown in Table 2. The configuration structure of sensors was tetrahedron, and the gyroscopes and accelerometers were installed coaxially, as shown in Figure 6. Therefore, the configuration matrixes of gyroscopes and accelerometers in RIMU are as follows:

(53) $$H^{g}=H^{a}=\left[\begin{array}{ccc} 0 & 0 & -1\\ \sin\alpha & 0 & \cos\alpha \\ \sin\alpha \cos\beta_{3} & \sin\alpha \sin\beta_{3} & \cos\alpha \\ \sin\alpha \cos\beta_{4} & \sin\alpha \sin\beta_{4} & \cos\alpha \end{array}\right]$$

where $\alpha =70.53^{\circ }$, $\beta_{3}=120^{\circ }$, and $\beta_{4}=240^{\circ }$.

### 4.2. Simulation of Error Variation in Rotation

To demonstrate the correctness of the proposed dual-axis rotation scheme, the variation in RIMU errors in rotation was simulated according to the tetrahedron RIMU configuration of the RRINS prototype.

Due to the limitation of space, only one gyroscope error and accelerometer error were simulated. Considering the universality, we chose the third gyroscope and accelerometer, which are not parallel to the coordinate system axis, as the simulation objects.

The errors of the third gyroscope and accelerometer were as follows:

(1) Bias: $b_{3}^{g}=0.1^{\circ }/h$, $b_{3}^{a}=50\;\mu g$.
(2) Symmetric scale factor error: $\lambda_{3}^{g}=50\;ppm$, $\lambda_{3}^{a}=30\;ppm$.
(3) Asymmetric scale factor error: $\mu_{3}^{g}=50\;ppm$, $\mu_{3}^{a}=30\;ppm$.
(4) Installation error: $\delta_{u3}^{g}=\delta_{u3}^{a}=10^{\prime \prime }$, $\delta_{v3}^{g}=\delta_{v3}^{a}=10^{\prime \prime }$.

**Figure 7:**
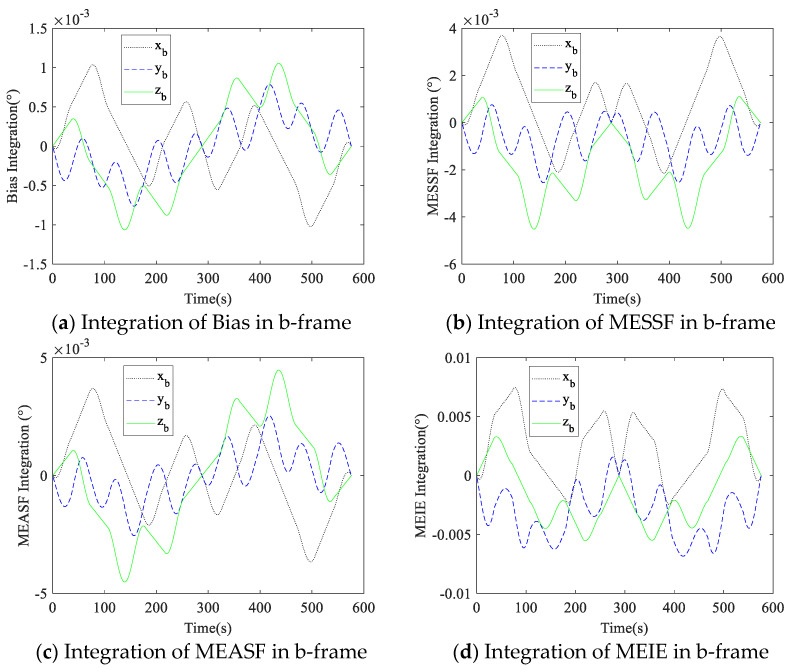
Integration of measurement errors caused by gyroscope errors in b-frame.

**Figure 8:**
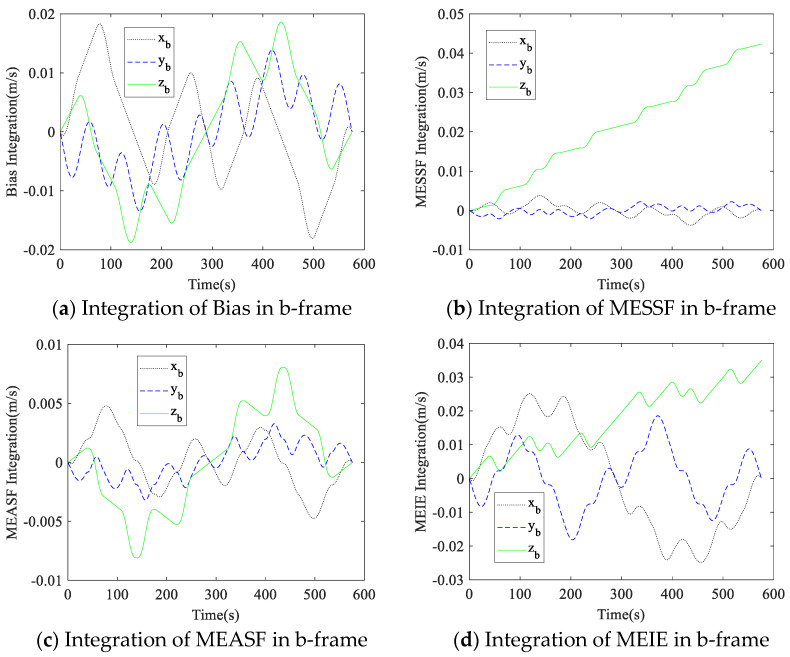
Integration of measurement errors caused by accelerometer errors in b-frame.

The measurement values of the gyroscopes and accelerometers were integrated into the calculation of inertial navigation, so the measurement errors diverged in the integration. In the simulation, the proposed dual-axis rotation scheme was applied, and the measurement errors caused by gyroscope and accelerometer errors in the b-frame were integrated. The variations in the measurement errors in the rotation are shown in Figure 7 and Figure 8.

It can be seen from Figure 7 that the projection of measurement errors caused by gyroscope errors in the b-frame were fully compensated after the 16-position rotation. The bias and MEASF of the accelerometer were fully compensated based on Figure 8, but the z-axis of MESSF and MEIE could not be compensated. The simulation results are consistent with the analysis in Section 2. Therefore, it can be seen that the dual-axis rotation scheme proposed in this paper is effective in compensating for the RIMU errors.

### 4.3. RIMU-Based and Traditional IMU-Based Navigation Simulation

Due to the redundant inertial sensor configuration of RIMU, the measurement data could not be directly used for strapdown navigation calculation. Therefore, it was necessary to use the weighted least squares method (WLS) to convert the redundant data of RIMU into the triaxial data in s-frame, as follows:

(54) $$\left\{\begin{array}{c} \omega^{s}={\left(H^{gT}W^{g}H^{g}\right)}^{-1}H^{gT}W^{g}N^{g}\\ f^{s}={\left(H^{aT}W^{a}H^{a}\right)}^{-1}H^{aT}W^{a}N^{a} \end{array}\right.$$

where $W^{g}$ and $W^{a}$ are the WLS weights of the gyroscopes and accelerometers, respectively.

After obtaining the triaxial inertial data of the RIMU in the s-frame, the navigation calculation could be carried out according to the traditional strapdown inertial navigation calculation method. Because of the characteristics of redundant data in RIMU, choosing appropriate $W^{g}$ and $W^{a}$ can make the navigation accuracy of RIMU better than that of IMU composed of the same level of inertial sensors.

#### 4.3.1. Strapdown Navigation Simulation

The strapdown inertial navigation calculations of RIMU and IMU were simulated to verify the superiority of RIMU redundant data. In the simulation, the inertial measurement data of IMU and RIMU in the static state were simulated and used in the calculation of inertial navigation. For the convenience of analysis, only the biases of gyroscopes and accelerometers were simulated.

The errors of IMU are as follows:

(1) All gyroscopes in IMU have a bias: 0.1°/h.
(2) All accelerometers in IMU have a bias: 50 μg.

Due to the particularity of the tetrahedral configuration, if the biases of all gyroscopes and accelerometers are the same, this will completely cancel out the biases, which is not conducive to our analysis. In fact, the actual application of inertial sensors cannot have exactly the same biases. Therefore, the bias of each sensor in RIMU was set to be slightly different. The errors of RIMU were as follows:

(1) The biases of four gyroscopes in RIMU: 0.1°/h, 0.11°/h, 0.12°/h, 0.13°/h.
(2) The biases of four accelerometers in RIMU: 50 μg, 55 μg, 60 μg, 65 μg.

**Figure 9:**
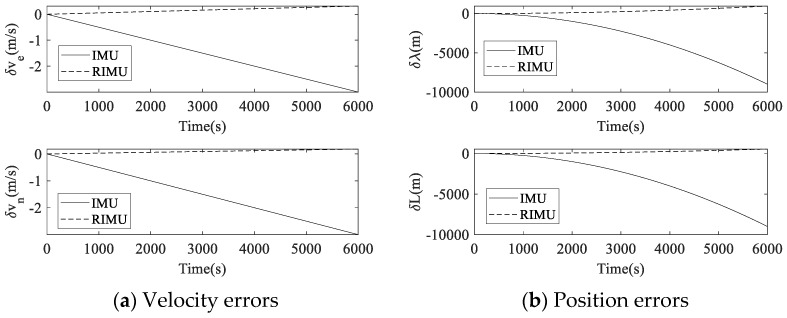
Strapdown navigation error comparison between IMU and RIMU. Here, $\delta v_{e}$ is the eastward velocity error, $\delta v_{n}$ is the northward velocity error, δλ is the longitude direction position error, and δL is the latitude direction position error.

The strapdown navigation calculation of IMU and RIMU in the static state was carried out, and the navigation errors are shown in Figure 9.

During 6000 s of navigation, the maximum velocity error of IMU was 3 m/s in both eastward and northward directions. In contrast, the maximum eastward and northward velocity errors of RIMU were 0.32 m/s and 0.18 m/s, respectively. The maximum position error of IMU was 9 km in both longitude and latitude directions, and the maximum longitude and latitude direction position errors of RIMU were 0.95 km and 0.55 km, respectively. It can be seen that the navigation accuracy of RIMU is much better than that of IMU by using the same level of inertial sensors.

#### 4.3.2. Rotational Navigation Simulation

**Figure 10:**
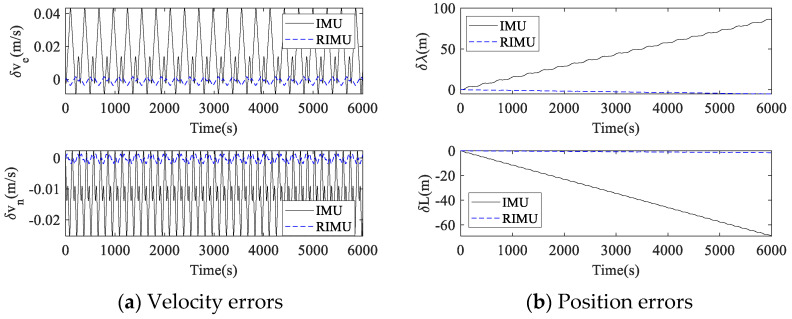
Rotational navigation error comparison between IMU and RIMU.

The proposed dual-axis rotation scheme was applied to the simulated IMU and RIMU, and the navigation calculation of the rotating IMU and RIMU was carried out. The error settings of IMU and RIMU were consistent with those of Section 4.3.1, and the navigation errors are shown in Figure 10.

After the proposed rotation scheme was applied, the velocity errors of IMU and RIMU no longer diverged, but showed a trend of periodic change. However, the velocity error fluctuation amplitudes of RIMU were much smaller than those of IMU. The fluctuation amplitudes of the eastward and northward velocity errors of IMU were 0.05 m/s and 0.03 m/s, while those of RIMU were 0.005 m/s and 0.003 m/s, respectively. The maximum longitude and latitude direction position errors of IMU were 86 m and 69 m, while those of RIMU were 5 m and 2 m, respectively. Therefore, RIMU-based RINS is superior to traditional IMU-based RINS in performance. In addition, it can be seen from Figure 9 and Figure 10 that the velocity errors and position errors of the rotational IMU and RIMU were much smaller than those of the strapdown IMU and RIMU.

### 4.4. Static Experiment

**Figure 11:**
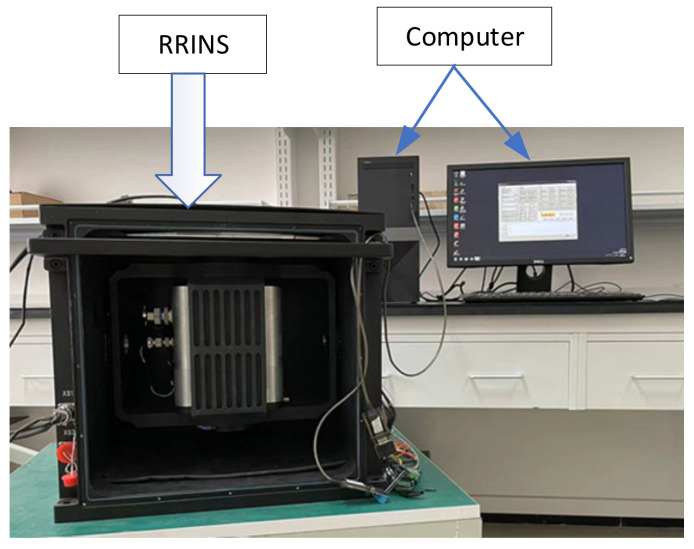
RRINS experimental setup.

A static experiment was performed using the RRINS prototype to demonstrate the improvement in RRINS navigation after applying the proposed dual-axis rotation scheme. The experimental setup of the RRINS is shown in Figure 11, and the other parameters of the experiment were as follows:

1 The RRINS remained stationary throughout the experiment.
2 Initial longitude: 116.668° E, latitude: 40.3554° N, height: 40 m.
3 Initial velocity: 0.
4 Dual-axis rotation parameters: angular rate of rotation: 2°/s, one position period: 100 s (rotating 90 s and standing 10 s), 16-position period: 1600 s.

**Figure 12:**
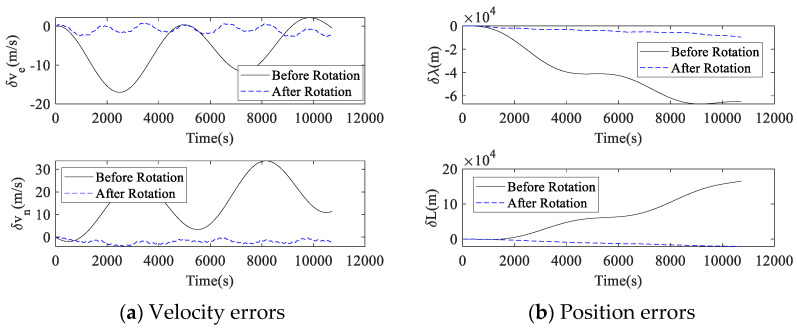
Navigation errors before and after rotation.

First, the turntable was controlled to keep the RIMU stationary for about 3 h for strapdown navigation calculation. Then, the turntable was controlled to rotate the RIMU according the proposed 16-position dual-axis rotation scheme for about 3 h, and the navigation calculation was performed. The whole experiment took about 6 h. After the experiment, the navigation velocity was taken as the velocity error, and the comparison of the velocity errors before and after rotation is shown in Figure 12a. The navigation position errors were calculated by subtracting the initial position from the whole navigation position, and the comparison of the position errors before and after rotation is shown in Figure 12b.

In the strapdown navigation experiment, the absolute value of RRINS velocity error in the east was up to 17.0 m/s, and velocity error in the north was up to 33.7 m/s. After applying the proposed dual-axis rotation scheme, the maximum absolute velocity error in the east was 2.65 m/s, and that in the north was 4.27 m/s. In terms of position errors, the maximum absolute values of the longitude and latitude direction position errors were 69 km and 192 km before rotation, respectively. After the rotation was performed, the maximum absolute values of the longitude and latitude direction position errors were 9.6 km and 21.3 km, respectively. It can be seen that the velocity and position errors of RRINS in the rotation case are much smaller than those in the strapdown case. After rotation, RRINS navigation accuracy was improved several times, and the effect is relatively obvious.

### 4.5. Dynamic Semi-Physical Simulation

**Figure 13:**
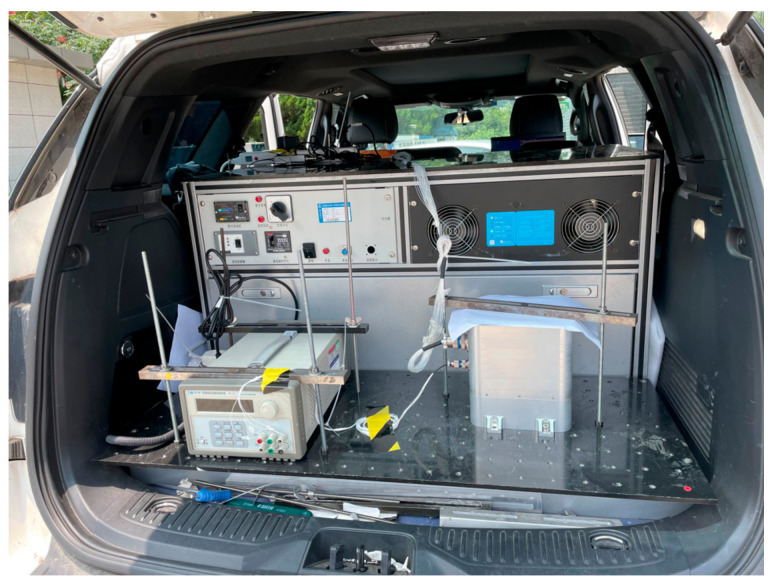
Data acquisition of vehicle motion.

To further demonstrate the comprehensive performance of the proposed system construction method, a dynamic semi-physical simulation was performed. The vehicle motion data collected by the research group before were examined, and the dynamic performance of RRINS was simulated based on these actual vehicle data. A high-precision integrated navigation system installed on the vehicle was responsible for collecting the angular rate, acceleration, attitude, velocity, and position of the vehicle. The experimental vehicle is shown in Figure 13.

**Figure 14:**
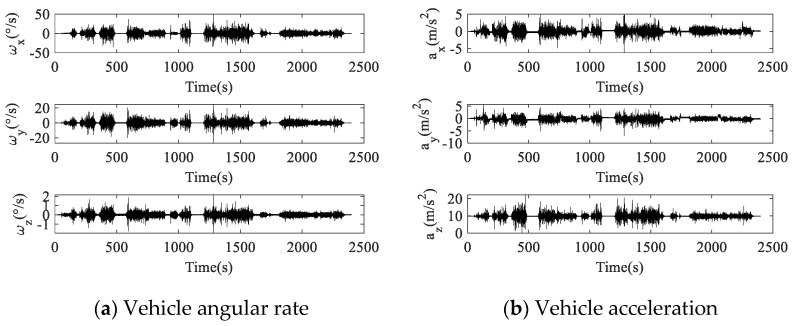
Motion data of vehicle.

**Figure 15:**
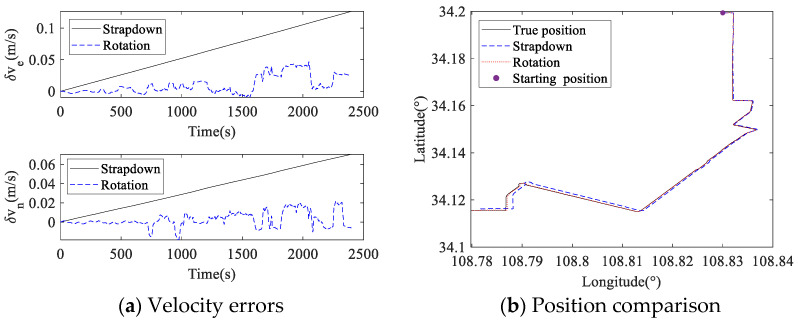
Navigation error comparison between strapdown and rotation in vehicle motion.

The angular rate and acceleration of vehicle are shown in Figure 14. The angular rate and acceleration of the vehicle were converted to the RIMU s-frame using the attitude conversion information, and the measurement data of the RIMU inertial sensors were simulated. The settings of RIMU errors were the same as described in Section 4.3. The navigation process of RIMU in the strapdown and rotation state was simulated, and the navigation error comparison between strapdown and rotation RIMU in vehicle motion is shown in Figure 15.

The vehicle velocity and position collected by the high-precision integrated navigation system are considered to be the true velocity and position of the vehicle. In less than 2500 s of vehicle motion, the absolute value of strapdown RIMU velocity error in the east was up to 0.13 m/s, and velocity error in the north was up to 0.07 m/s. For rotation RIMU, the maximum absolute velocity error in the east was 0.05 m/s, and that in the north was 0.02 m/s. It can be seen from the position navigated by strapdown RIMU and rotation RIMU that the position navigated by rotation RIMU was obviously closer to the true position than that navigated by strapdown RIMU. The end point navigated by strapdown RIMU was 195 m from the real end point, while that by rotation RIMU was 30 m. Therefore, the proposed dual-axis rotation scheme in RRINS also has advantages in dynamic vehicle navigation.

## 5. Conclusions

To the best of our knowledge, this is the first study to develop a dual-axis rotation scheme based on the RIMU error model in the RRINS. The general inertial sensor errors and installation errors in RIMU were modeled and expressed in vector form. The variation characteristics of gyroscope and accelerometer errors in RRINS rotation were analyzed in detail based on the RIMU error model. On the basis of the characteristic analysis of error variation, the principles of rotation axis switching and reciprocating rotation were summarized. According to the rotation axis switching principle and the reciprocating rotation principle, and considering the characteristics of RIMU errors, a 16-position dual-axis rotation scheme was designed. The designed rotation scheme can effectively compensate the bias, installation error, symmetric scale factor, and asymmetric scale factor error of gyroscopes and accelerometers in RIMU in real time. Simulations and experiments were performed after the RRINS prototype was constructed, and the results are summarized in Table 3.

The results of the simulations and experiments show that the position navigation errors of RRINS were greatly reduced after the proposed method was applied, thus proving the effectiveness and superiority of the proposed dual-axis rotation scheme.

## Figures and Tables

**Table 1:** The 16-position dual-axis rotation scheme.

Position	Rotation of RIMU		Control of Turntable	
	Axis	Angle	Axis	Angle
1	*z*	+180°	Inner	+180°
2	*x*	+180°	Outer	−180°
3	*z*	+180°	Inner	+180°
4	*x*	+180°	Outer	+180°
5	*x*	+180°	Outer	+180°
6	*z*	+180°	Inner	+180°
7	*x*	+180°	Outer	−180°
8	*z*	+180°	Inner	+180°
9	*z*	−180°	Inner	−180°
10	*x*	−180°	Outer	+180°
11	*z*	−180°	Inner	−180°
12	*x*	−180°	Outer	−180°
13	*x*	−180°	Outer	−180°
14	*z*	−180°	Inner	−180°
15	*x*	−180°	Outer	+180°
16	*z*	−180°	Inner	−180°

**Table 2:** Parameters of gyroscopes and accelerometers in RIMU.

Parameter	Gyroscope	Accelerometer
Range	−300 to + 300 °/s	−20 to + 20 g
Bias	−10 to + 10 °/h	≤3 mg
Stability (4 in order)	[0.0502, 0.0303, 0.0355, 0.0517] °/h	[12.4, 8.3, 27.7, 11.7] μg
Repeatability (4 in order)	[0.0831, 0.0109, 0.0720, 0.0770] °/h	[5.7, 5.8, 7.4, 5.1] μg
Scale Factor Repeatability	≤50 ppm	≤30 ppm

**Table 3:** Results of simulations and experiments.

Object	Static Simulation		Static Experiment		Dynamic Semi-Physical Simulation
	$\max\left|\delta \lambda \right|$	$\max\left|\delta L\right|$	$\max\left|\delta \lambda \right|$	$\max\left|\delta L\right|$	End Point Error
Strapdown IMU	9 km	9 km	-	-	-
Rotation IMU	86 m	69 m	-	-	-
Strapdown RIMU	0.95 km	0.55 km	69 km	192 km	195 m
Rotation RIMU	5 m	2 m	9.6 km	21.3 km	30 m

## Data Availability

Not applicable.
